# Peptide delivery with poly(ethylene glycol) diacrylate microneedles through swelling effect

**DOI:** 10.1002/btm2.10070

**Published:** 2017-07-14

**Authors:** Shiying Liu, David C. Yeo, Christian Wiraja, Hong Liang Tey, Milan Mrksich, Chenjie Xu

**Affiliations:** ^1^ School of Chemical and Biomedical Engineering Nanyang Technological University 70 Nanyang Drive, 637457, Singapore; ^2^ National Skin Centre 1 Mandalay Road, 308205, Singapore; ^3^ Lee Kong Chian School of Medicine Nanyang Technological University 50 Nanyang Avenue, 639798, Singapore; ^4^ Dept. of Chemistry Northwestern University 2145 Sheridan Road, Evanston, 60208 IL; ^5^ NTU‐Northwestern Institute for Nanomedicine Nanyang Technological University 50 Nanyang Avenue, 639798, Singapore

**Keywords:** microneedles, keloid scar treatment, transdermal drug delivery, peptide, (polyethylene glycol) diacrylate

## Abstract

Transdermal delivery of therapeutic biomolecules (including peptides) can avoid enzymatic digestion that occurs in the oral route. (Polyethylene glycol) diacrylate (PEGDA)‐based microneedles, with good biocompatibility, are easily fabricated through photo‐polymerization with a precisely controlled structure. It has successfully been used for the transdermal delivery of small molecule drugs such as 5‐fluorouracil. However, the delivery of peptide‐based therapeutics using this platform is seldom reported. This is because of the potential damage to the peptide during the photo‐polymerization process of PEGDA. Herein, we introduce a method to load PEGDA microneedles with peptides without compromising peptide potency. Using gap junction inhibitor (Gap 26) as an example, the peptide was loaded into PEGDA microneedles through the swelling effect of PEGDA in the aqueous solution. The peptide‐loaded microneedles were applied to a keloid scar model and exhibited inhibition expression of collagen I, a predominant marker of keloid scar, demonstrating its potential therapeutic effects.

## 1 INTRODUCTION

Microneedles are emerging as a proficient transdermal delivery system. It allows a variety of molecules to be transported into skin in a minimally invasive way, which overcomes the limitations of conventional needle injection.^1^, ^2^, ^3^ Polymer‐based microneedles are specifically attractive due to their excellent biocompatibility, biodegradability, and nontoxicity.^4^, ^5^, ^6^ Drugs can be loaded into the microneedle tips and/or base, or coated on the microneedle tips.^1^, ^4^, ^5^ One example is poly(ethylene glycol) diacrylate (PEGDA)‐based microneedles.^7^, ^8^, ^9^ PEGDA is biocompatible and can be cross‐linked in a short time under UV exposure (a few seconds), which facilitates the control of morphology and dimension of microneedles.^7^, ^10^ Small molecule drugs like 5‐fluororacil (5‐FU) and curcumin have been integrated within microneedles during the fabrication process,^11^ in which drugs are mixed with the monomer before the mixture is placed in a mold and exposed to UV light. For example, our group has utilized PEGDA‐made microneedles to deliver the 5‐FU for treating keloid scar cells.^12^ PEGDA microneedles have sufficient mechanical strength to penetrate skin and release drugs to inhibit keloid fibroblast growth. In addition, other small molecule drugs, such as hydrophobic campothecin (CPT)—that targets DNA topoisomerase I (Topo I) and inhibit collagen synthesis^13^ can also be encapsulated and delivered in a similar manner.

In recent years, peptide‐based therapeutics have played a major role in new drug development.^14^, ^15^, ^16^, ^17^ However, this process has not been without difficulties. Peptides face hurdles including instability under high temperature, light, and high/low pHs, and digestion by gastrointestinal enzymes during oral delivery. Microneedles have been suggested as a means to overcome this delivery hurdle since it can bypass oral uptake routes. The easiest way to load the peptide is to coat them on the surface of needles, however, it often requires optimization and adjusting the coating formulations by adding excipients to increase viscosity and decrease surface tension in order to achieve uniform and sufficient coating.^18^ Preloading drugs into the needles during the fabrication is an alternative way. However, preloading processes should not disrupt or significantly compromise peptide activity. PEGDA microneedles have been used to deliver several small molecular drugs that are preloaded into the polymer matrix during the fabrication. However, this procedure is unsuitable for peptides since high energy UV rays can denature peptides thus compromise the biological activity of peptide‐based therapeutics.^19^, ^20^

**Figure 1:**
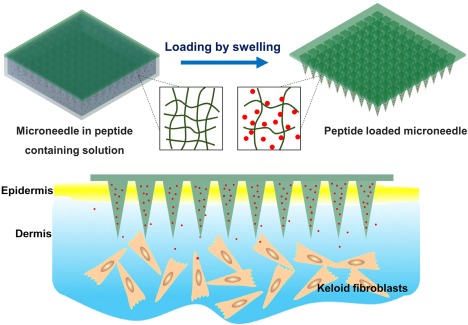
Schematic showing the loading of hydrophilic peptides into poly(ethylene glycol) diacrylate microneedles by the swelling effect

This report introduces a gentle strategy to load peptides into the PEGDA microneedles without the above complications based on the swelling ability of PEGDA in aqueous solution. We discovered that substances with molecular mass less than 4 kDa readily enter the PEGDA matrix during swelling in aqueous solutions. Furthermore, this process is dependent on the period of UV exposure to crosslink PEGDA monomers (part of the microneedle fabrication process). As a proof of concept, the model peptide (Gap 26, a connexin mimetic peptide that inhibits cellular gap‐junction),^21^, ^22^, ^23^, ^24^, ^25^, ^26^, ^27^, ^28^ is loaded into the PEGDA microneedles using this PEGDA swelling strategy (Figure 1). After examining the loading capacity and release profile of the peptide‐loaded microneedles, the potential therapeutic effect is explored on a keloid scar model comprising of cells and ex vivo skin.

## 2 MATERIALS AND METHODS

### 2.1 Materials

Fluorescein Diacetate (FDA), Calcein‐AM, Vybrant® DiI Cell‐Labeling Solution, and Geltrex™ LDEV‐Free Reduced Growth Factor Basement Membrane Matrix were purchased from Thermo Fisher Scientific Inc. Goat Anti‐Mouse IgG (H + L) Antibody and Rhodamine conjugate were from Sigma Aldrich. Keloid fibroblasts (KF110) were ordered from Cell Research Corporation (Singapore). Dulbecco's modified eagle medium (DMEM) (w/4.5 g/L d‐glucose, w/phenol red), fetal bovine serum (FBS), penicillin‐streptomycin (10,000 U/ml), Phosphate buffered saline (1x, PBS), and trypsin‐EDTA (0.25%, 10x) were obtained from Gibco Life Technologies (USA). All reagents were of analytical reagent grade and used without further purification.

### 2.2 Fabrication of blank and CPT loaded PEGDA microneedles

First, a negative polydimethylsiloxane (PDMS) mold of microneedles was prepared by curing PDMS (base: curing agent = 10:1) on the stainless microneedle template for 3 hr at 70°C. In order to make blank PEGDA microneedles, the mixed solution of PEGDA (MW = 250) and photoinitiator 2‐hydroxy‐2‐methylpropiophenone (0.5% v/v) was added to the negative PDMS mold pretreated with plasma. The gas bubbles in the solution were removed by vacuum before the whole sample was exposed to UV (Intensity: 10 mW/cm^2^) for crosslinking. Finally, PEGDA microneedles patch was peeled off from the PDMS mold.

The CPT loaded PEGDA microneedles were fabricated in a similar way as the blank microneedles except that the CPT was present in PEGDA solution. CPT loaded microneedles were imaged by confocal laser scanning microscope (Carl Zeiss, LSM 710) with excitation at 405 nm laser and emission at 410–585 nm. Mechanical properties of microneedles were measured by Instron 5543 Tensile Meter with a speed of 0.5 mm/min.

### 2.3 Swelling ratio and mesh size determination

The microneedle patches were placed in distilled water for 22 hr. Then the samples were taken out and gently dried on the paper, and the weight in the swollen state (*W*_swell_) was obtained. Later, the patches were transferred into an oven preheated to 50°C and dehydrated until a stable dry weight (*W*_dry_). The swelling ratio can be calculated by Equation (1).

(1) $$Q=\frac{W_{swell}-W_{dry}}{W_{dry}}\;\times \;100\%$$

To calculate the mesh size,^29^ the polymer volume fraction of the swollen state *υ_2s_* was first obtained by Equation (2).

(2) $$\upsilon_{2s}=\frac{1}{Q\frac{\rho }{\rho_{H_{2O}}}+1}$$

where *Q* is the swelling ratio, ρ is polymer density, and $\rho_{H_{2O}}$ is water density.

The average molecular weight between two consecutive crosslinks ( $M_{c}$) can be calculated by Equation (3), where $M_{n}$ is the average molecular weight of the starting polymer (250 g/mol) here, $V_{1}$ is the molar volume of water (18 cm^3^/mol), χ is the Flory‐Huggins polymer solvent interaction parameter and assumed equal to 0.426, $\upsilon_{2r}$ is the polymer volume fraction in the relaxed state (i.e., right after crosslinking and before full swelling), and the polymer volume fraction in the precursor solution was assumed as indicative of $\upsilon_{2r}$.

(3) $$\frac{1}{M_{c}}=\frac{2}{M_{n}}-\frac{\frac{1}{V_{1}}\left[ln\left(1-\upsilon_{2s}\right)+\upsilon_{2s}+\chi {\upsilon_{2s}}^{2}\right]}{\rho \upsilon_{2r}\left[{\left(\frac{\upsilon_{2s}}{\upsilon_{2r}}\right)}^{\frac{1}{3}}-\frac{1}{2}\left(\frac{\upsilon_{2s}}{\upsilon_{2r}}\right)\right]}$$

Finally, mesh size can be calculated by the Equation (4).

(4) $$\xi =l{\left(2\frac{M_{c}}{M_{r}}\right)}^{\frac{1}{2}}{C_{n}}^{\frac{1}{2}}{\upsilon_{2s}}^{-\frac{1}{3}}$$

where *l* is the bond length (1.5 A^o^), *M*_r_ is the molecular weight of the PEG repeating unit (44 g/mol), and *C*_n_ is the characteristic ratio for PEG, equal to 4.

### 2.4 Fabrication of hydrophilic molecules (FITC, FITC‐Dextran, Gap 26) loaded PEGDA microneedles

Aqueous solutions containing FITC and FITC‐Dextran with different molecular weight (i.e. 4, 10, 20, and 70 kDa) were prepared with DI water and diluted to have the same FITC fluorescence intensity. A specific reservoir with flat bottom was made similarly by PDMS using the stainless microneedles to allow one patch of microneedles to be immersed in the hydrophilic solution (100 μl) for loading. After 22 hr incubation, the microneedle patch was washed with water twice to remove the molecules on the surface before being dried naturally. The FITC and FITC‐Dextran loaded microneedle patches were imaged by confocal laser scanning microscope (Carl Zeiss, LSM 710) with excitation at 488 nm laser and emission at 493–634 nm.

FITC‐labeled Gap 26 (FITC‐C6‐VCYDKSFPISHVR, MW = 2,053.3) and unlabeled Gap 26 (VCYDKSFPISHVR, MW = 1550.8) were acquired from Sigma. Similar to the loading of FITC and FITC‐Dextran, Gap 26 was loaded into blank PEGDA microneedles through the incubation in the reservoir for 22 hr. FITC conjugated Gap 26 loaded microneedles were imaged by confocal laser scanning microscope (Carl Zeiss, LSM 710) with excitation at 488 nm laser and emission at 493–634 nm. The loading efficiency was calculated by comparing the total amount of peptides and the remaining in solution.

### 2.5 Release profile of CPT and Gap26 from microneedles

Microneedle patches loaded with drugs were immersed in DI water at 37°C on a shaking device with a speed of 500 rpm. At desired time points, 50 μl of solution was transferred to Greiner 96 well plate and the fluorescence intensity was examined with Synergy H4 (CPT: excitation 365 nm/emission 428 nm; FITC‐Gap 26: excitation 495 nm/emission 525 nm). The concentrations were calculated based on standard curves and cumulative released amount of drugs were calculated for different time points.

### 2.6 Cell viability test by AlamarBlue® assay

Keloid fibroblasts were seeded in 96 well plate at a density of 1,500 cells per well. After overnight culturing, culture medium was replaced with new culture medium containing different concentrations of free CPT (0.1 μM, 10 μM) for 24 hr. After 24 hr, the medium was removed and cells were gently washed by PBS for three times. Finally, cell medium with AlamarBlue reagent was added. Four hours later, fluorescence of the solution (emission/excitation: 555/585 nm) was examined and analyzed according to manufacturer's protocol. All the data were normalized by taking the viability of untreated cells as 100%.

### 2.7 FRAP assay

Keloid fibroblasts were seeded at 24 well plate at a density of 40,000 cells per well. Once they reached confluence, cells were incubated with serum‐free medium for starvation for 1 hr. Then medium was replaced with serum‐free medium containing different concentrations of Gap 26 (0.125, 0.25, 0.5 mg/ml) for 2 hr. Later, cells were stained with calcein‐AM for 20 min. After washing twice by PBS, cells were imaged through confocal laser scanning microscope (Carl Zeiss, LSM 710) with excitation at 488 nm and emission at 493–616 nm. Thereafter, fluorescence recovery after photobleaching (FRAP) assay was performed at a selected specific region with 50% of 488 nm laser intensity to bleach the fluorescence for three cycles and immediately continuous imaged for 20 min to record the fluorescence recovery (imaged once per minute).

### 2.8 Real‐time polymerase chain reactions for collagen I expression

Keloid fibroblasts were seeded at the 24 well plate at a density of 40,000 cells per well. Once they reached confluence, culture medium was replaced with culture medium with different concentrations of free CPT (0.1, 10 μM) for 24 hr. Later cells were washed by PBS for three times before being lysed in Lysis buffer with β‐mercaptoethanol. Subsequently, RNA was extracted by RNAeasy mini kit and the concentration was detected by NanoDrop UV‐vis Spectrophotometer. The complementary DNA was synthesized according to the templates of RNA by Bioline SensiFAST cDNA Synthesis Kit. Finally, in order to quantify the gene expression of samples, Real‐time Polymerase Chain Reactions (RT‐PCRs) were conducted with SYBR Green PCR Master Mix kit by Step One Plus Real‐Time PCR System. The process includes denaturation at 95°C for 10 min, and amplification for 40 cycles with 95°C denaturation for 15 s and 60°C extension for 1 min. The forward and backward primer sequences of glyceraldehyde 3‐phosphate dehydrogenase (GAPDH) and collagen I are listed in Supporting Information Table S1. The mRNA expression level of collagen I was normalized to the housekeeping gene GAPDH, and then calculated as fold change according to comparative CT method using the 2^−ΔΔCt^ formula.

### 2.9 3D agarose hydrogel model

About 400,000 keloid fibroblasts were suspended in 75 μl complete medium. Then, 75 μl of 3% agarose preheated for melting and cooled to 37°C was mixed with the cell suspension, in which the bubble formation was avoided. Subsequently, 10 μl of the suspension was added to each well of the 48 well plate. After being placed at 4°C for 4 min, the agarose gel formed and was placed in the incubator overnight. Later, CPT loaded PEGDA microneedles were inserted into agarose beads for 24 hr. After the removal of microneedles, cells were stained with FDA for 20 min and imaged with confocal microscope (LSM 710).

### 2.10 Ex vivo keloid model

Full‐thickness human skin derived from operations to remove excess tissue was purchased from Cell Research Corporation (Singapore). The skin samples were cut to 1.3 × 1.3 cm^2^ and maintained in complete medium (DMEM containing 10% FBS). During the maintenance in the incubator, the epidermis layer was exposed to the air while the dermis was submerged in the medium. In order to identify the location and distribution of injected cells, keloid fibroblasts were stained with DiI (1 ml complete medium with 3 μl DiI labeling solution) for 2 hr, followed by washing with PBS. Following overnight incubation, DiI‐labeled cells were trypsinized before resuspension in 100 μl cooled medium (15,000 cells per μl). The cell suspension was mixed with 100 μl GelTrex matrigel gently, to avoid the generation of bubbles. Thereafter, the cells—matrigel mixture was injected from the dermis‐side of the skin sample using a 30G needle. After resting at room temperature for 15 min, 200 μl of medium was added and the samples were incubated at 37°C overnight. After 24 hr, skin samples were fixed in formalin for 72 hr, dehydrated by 30% sucrose for 8 hr, and 4% sucrose for 18 hr. Finally, the samples were frozen in Tissue Freezing Medium and cryosectioned at a thickness of 10 μm by Leica CryoStat CM1950. The frozen slides were stored at −20°C before further characterization.

The therapeutic efficacy of drug‐loaded microneedles was examined with the skin samples injected with unlabeled keloid fibroblasts. The CPT and FITC loaded PEGDA microneedles were pressed into the skin sample by an applicator (Micropoint Pte Ltd). There were four groups: “CPT MN twice” (CPT MNs [low loading concentration: 18 nmol) were applied at day 14 and 21], “Gap 26 MN twice” (Gap 26 MNs [loading amount: 125 µg] were applied at day 14 and 21), “CPT MN once” (CPT MNs [low loading concentration: 18 nmol] were applied once at day 21), and “Gap 26 MN once” (Gap 26 MNs [loading amount: 125 µg] were applied once at day 21). At day 28, the patches were removed from all samples. The skin was fixed in formalin and processed as above for cryosection and immunostaining for collagen I examination.

### 2.11 Immunostaining for collagen I

The sliced skin samples were treated by 0.2% Triton‐X 100 in PBS for 10 min, followed by 0.1% Triton‐X 100 in PBS for 5 min. After being washed with PBS three times, they were placed in the 1% BSA in PBS for 30 min and labeled with primary antibody solution (mouse anti‐human collagen I, 1–100) at 4°C overnight. Later, the slides were washed by 0.1% Triton‐X 100 in PBS three times and stained with secondary antibody (Goat anti mouse IgG, Rhodamine conjugate, 1–100) at room temperature for 4 hr. Finally, Hoechst (10 μg/ml) was added to stain the nuclei. The stained slides were examined by confocal microscope (LSM 710) (Rhodamine: excitation at 561 nm and emission at 570–660 nm; Hoechst: excitation at 405 nm and emission at 410–543 nm).

### 2.12 Statistical analysis

All values were expressed as means ± standard deviation. One‐way ANOVA and Tukey HSD test was used for statistical analysis with *p* < .05 (*), *p* < .01 (**), and *p* < .001 (***) as significant.

## 3 RESULTS

### 3.1 PEGDA microneedles loaded with hydrophobic camptothecin

About 10 μM CPT was shown to inhibit the proliferation of keloid fibroblasts (Supporting Information Figure S1A) and suppress the collagen I expression (Supporting Information Figure S1B). Even only after 24 hr, the proliferative activity of CPT of fibroblasts was reduced by 18.21%. Simultaneously, the cellular collagen I expression per cell decreased ∼55% under the treatment.

As a hydrophobic molecule, CPT can be loaded into PEGDA microneedles during microneedle fabrication by dissolving it with PEGDA monomer. The PEGDA microneedles exhibited homogeneous pyramid‐like structures with a base diameter of 300 μm and a depth of ∼850 μm following photo‐polymerization (Supporting Information Figure S2A,B). The fluorescence from microneedles confirmed the presence of CPT (Supporting Information Figure S3A). The release profile of CPT was determined at desired time points by measuring fluorescence intensity and calculating the released amount based on a standard curve of predetermined drug concentrations (Supporting Information Figure S3B). Within 24 hr, 0.37 nmol CPT was demonstrated to be released. In 3D agarose gels containing keloid fibroblasts, CPT‐loaded microneedles similarly inhibited fibroblast proliferation (Supporting Information Figure S3C,D). Specifically, CPT loaded microneedles treated groups reduced cell proliferation by 30.1% compared to untreated cells in 24 hr.

### 3.2 Swelling effect of PEGDA microneedles in aqueous solution

**Figure 2:**
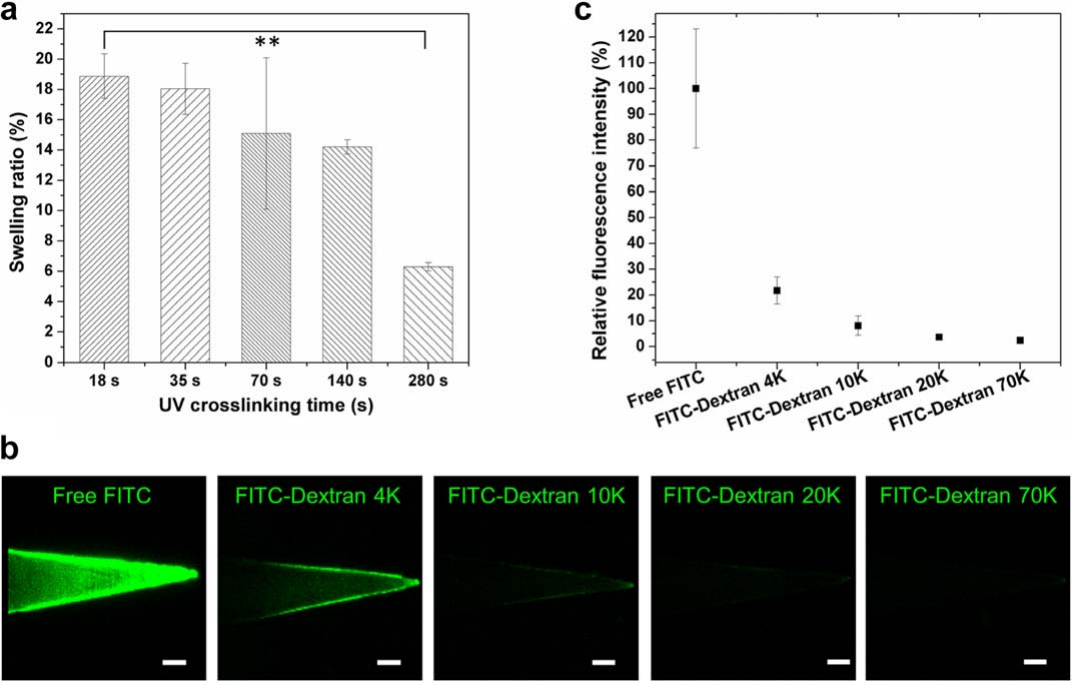
Swell performance of PEGDA microneedles and loading molecules with different molecular weight. (a) Swelling ratio of PEGDA microneedles prepared with different UV exposure time. **p < .01. (b) Confocal images of PEGDA microneedles loaded with FITC and FITC‐Dextran with different molecular weight. Scale bar: 100 μm. (c) Quantified microneedle fluorescence intensity in B relative to that with free FITC

PEGDA exhibits swelling behavior in aqueous solutions.^29^ As shown in Figure 2a, PEGDA microneedles fabricated using different UV exposure exhibited different swelling properties. Generally, longer UV exposures resulted in smaller swelling ratios (*Q*). Microneedles were observed to increase their volume by up to 18.88%. Mesh size can be calculated using the Equations (1), (2), (3), (4). A higher swelling ratio suggests a larger mesh size (Supporting Information Figure S4). Specifically, the mesh size was 2.0 nm when the patch was prepared with a short UV exposure time (18 s). PEGDA microneedle patch crosslinked with UV exposure for a period of 18 s was selected for the subsequent experiments.

In the swelling process, molecules like FITC and FITC‐Dextran can enter the polymer matrix through diffusion (Figure 2b). Obviously, this process is size‐dependent, with smaller molecules enter the polymer matrix more readily. In addition, the distribution of loaded FITC and FITC‐Dextran molecules within microneedles was inhomogeneous, with more observed at the edge. Increased cargo size (4–70 kDa), led to a reduction in loading efficiency as reflected in a decrease in microneedle fluorescence signal (Figure 2b,c). Above 4 kDa, the molecules exhibit poor diffusion into the polymeric matrix. Based on the total fluorescence signal from microneedle samples, their relative loading efficiency (compared to free FITC) was found to be 21.7, 8.1, 3.7, and 2.4% for FITC‐Dextran 4, 10, 20, and 70 kDa, respectively.

### 3.3 Peptide loaded PEGDA microneedles

Since the characterization of PEGDA swelling showed that hydrophilic molecules smaller than 4 kDa can be loaded into microneedles, we hypothesized that the peptide Gap 26 (MW = 1550.8) could similarly be loaded. Gap26, as a representative connexin mimetic peptide, can mimic specific amino acid sequences of two well‐conserved extracellular loops of connexins and interfere with their functions. Gap junctions are formed by intercellular channels composed of connexin proteins, which are known to impact inflammatory response, would closure, and scar formation.^23^, ^27^, ^28^, ^30^, ^31^ Suppressing connexins activity by antisense oligodeoxynucleotide was found to reduce the spread of tissue damage, accelerate wound closure, and reduce scarring.^32^ So, we hypothesized Gap 26 is able to disturb connexins functions, interfere with gap junction‐based intercellular communications, thus exhibit potential for keloid scar treatment.

**Figure 3:**
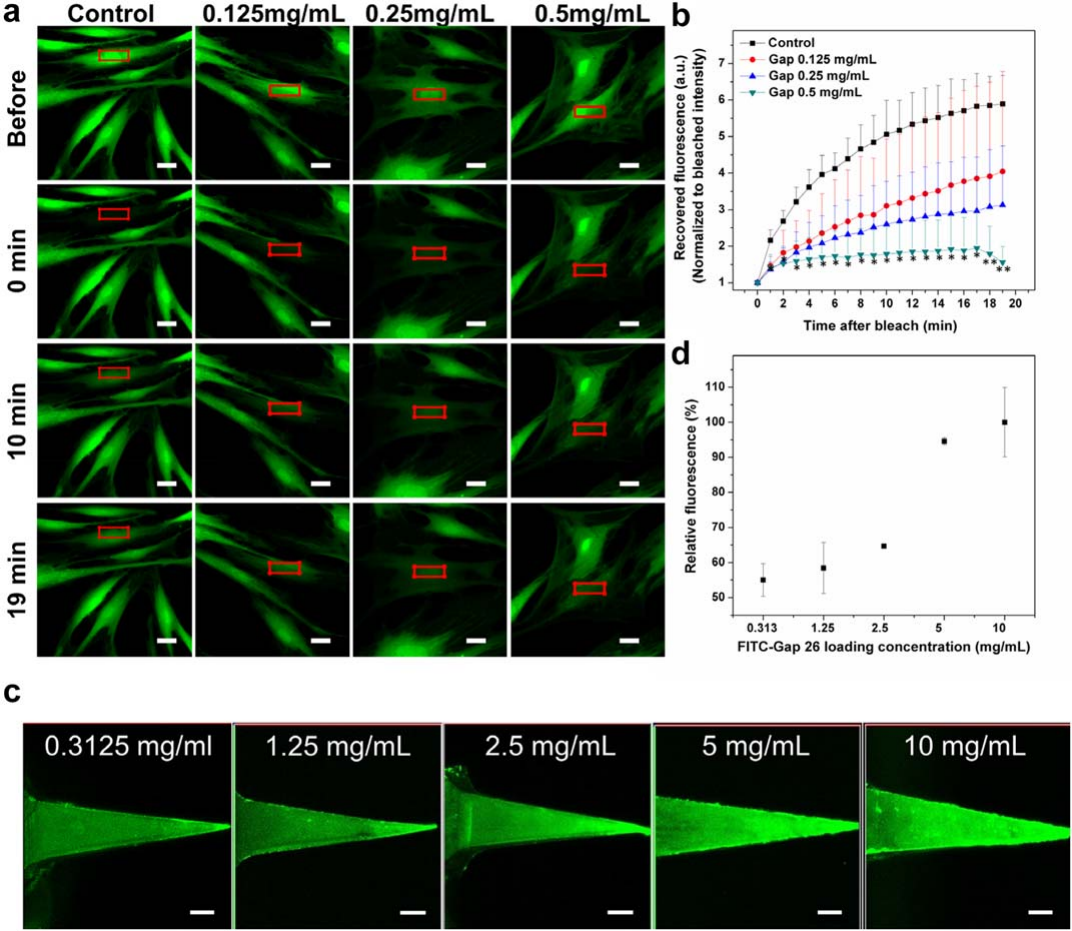
Effect of Gap 26 on FRAP of keloid fibroblasts and loading of FITC‐Gap 26 into PEGDA microneedles. (a) Confocal images of keloid fibroblasts in FRAP experiment with the treatment of Gap 26. Scale bar: 20 μm. (b) Recovered fluorescence intensity change during fluorescence recovery. *p < .05, **p < .01. (c) Confocal images of Gap26 loaded PEGDA microneedles. Scale bar: 100 μm. (d) Quantitative analysis of C

FRAP assay was performed to characterize the inhibitory effect of Gap 26 in gap junction‐based intercellular communication.^33^ Fibroblasts were initially incubated with Gap26 before calcein staining. Subsequently, selected area of the cells was photobleached using high power laser (the red box of Figure 3a) and the recovery of calcein fluorescence at that region was recorded (Figure 3b). The fluorescent intensity during the recovery period was compared with the intensity immediately after bleaching. In 20 min, the fluorescence of untreated cells increased to 5.89 folds. However, the presence of Gap 26 peptide significantly delayed this recovery. For example, only a 1.56‐fold increase in the fluorescence intensity was observed in keloid fibroblasts treated with 0.5 mg/ml Gap 26. This suggests considerable attenuation of intercellular communication by gap junctions, indicating the efficacy of Gap26 against connexins, intercellular channels, showing promise for the improvement of scar treatment.

Subsequently, we loaded PEGDA microneedles with Gap26 through the swelling effect of PEGDA. Gap26 was labeled with FITC to facilitate quantification. As shown in Figure 3c,d, a higher peptide concentration in the incubation process provided a higher peptide loading in the microneedles. By quantifying the peptides in the loading solution before and after incubation, the loading efficiency of peptide was calculated (Supporting Information Table S2). For example, 252.13 ± 5.94 μg of Gap26 can be loaded to one patch of microneedles (10 by 10 tips) with 50.43 ± 1.19% loading efficiency for the initial loading amount of 500 μg. As the loading process was performed with only the microneedle tips immersed inside the hydrophilic solution, the drugs were only localized in the tips without excess drug trapped in the base. This maximizes the utilization of the drugs. In this example, each tip was loaded with 2.52 ± 0.06 μg of Gap26.

The loaded peptides were promptly released once the microneedles were placed in the aqueous solution. As shown in Supporting Information Figure S5, 13.47 ± 4.52 μg of FITC‐Gap 26 can be released from the tips of microneedles that contained 252.13 ± 5.94 μg peptides in total.

### 3.4 Therapeutic effect of Gap26 loaded microneedles in ex vivo keloid model

**Figure 4:**
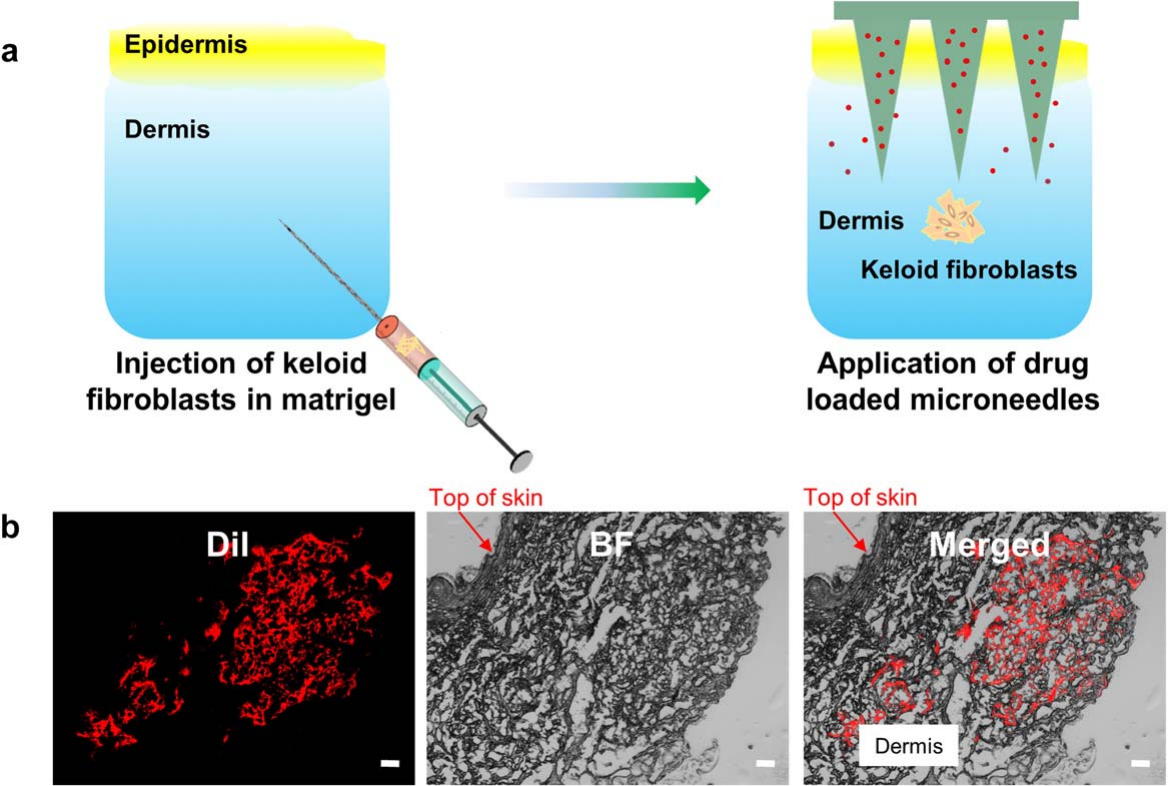
Ex vivo keloid scar model and injection of keloid fibroblasts. (a) Illustration of the ex vivo keloid model. (b) Presence of DiI labeled keloid fibroblasts in the dermis region of skin samples. Scale bar: 100 μm

A keloid disease model was next used to explore the potential efficacy of Gap26‐loaded microneedles. Briefly, the model was built by injecting keloid fibroblasts into the dermis region of an ex vivo human skin sample (Figure 4a). Keloid fibroblasts were labeled with the lipophilic dye DiI (Supporting Information Figure S6) to enable identification (Figure 4b).

Before the study commenced, keloid fibroblasts were injected into the dermis region of the skin. At day 14, the microneedle patch was applied on the skin for 1 week. The patch was then replaced with a new one at day 21 for another week to sustain drug release and maintain drug amount within the skin. We studied two types of microneedle patches, CPT‐, and Gap26‐ loaded microneedles. At day 28, skin samples were fixed and sectioned to examine the collagen I expression.

**Figure 5:**
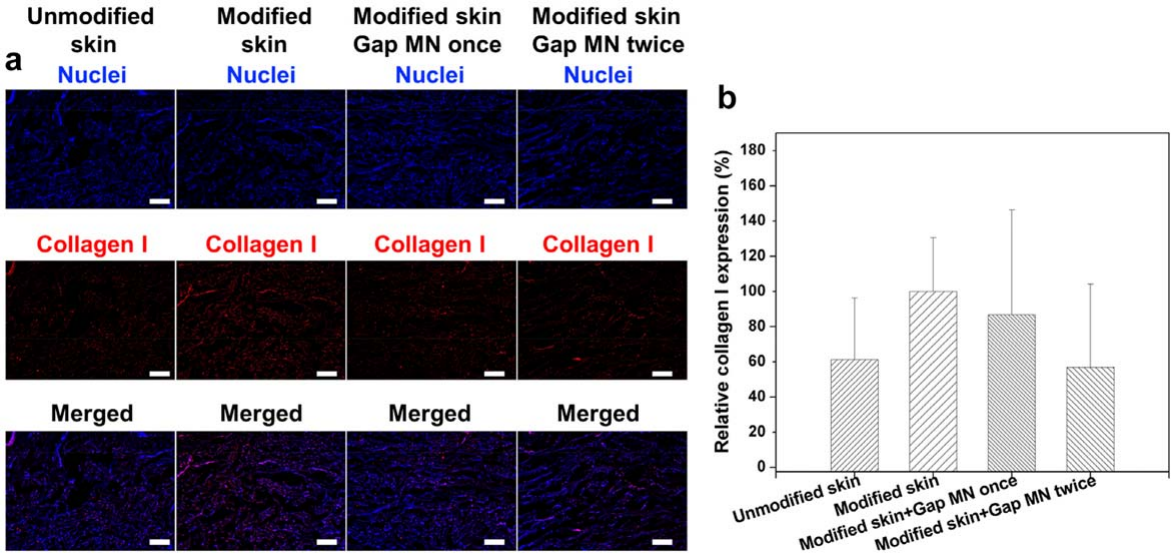
Effect of Gap 26 loaded microneedles on collagen I expression of skin samples in the ex vivo model. (a) Immunostaining of Collagen I expression after the treatment with Gap26‐loaded microneedles. Scale bar: 100 μm. (b) Quantitative analysis of collagen I expressions in A by normalization to the modified skin without treatment

The presence of keloid fibroblasts increased the expression of collagen I in the dermis layer of the skin (Figure 5a), which is reminiscent of native keloid scars.^34^, ^35^ Keloid scar is characterized by abundant deposition of collagen, especially collagen type I.^36^ The treatment with Gap 26 loaded microneedles twice (“Gap 26 MN twice”) reduced the collagen I expression to 36.9% (Figure 5b). Treatment with CPT loaded microneedles twice (“CPT MN twice”) also suppressed collagen I expression to 47.3% (Supporting Information Figure S7). The PEGDA microneedles still maintained their homogeneous pyramid tips structure after application in the skin samples and did not dissolve or deform in the skin to leave any remnants as shown in Supporting Information Figure S8. These results indicated the CPT and Gap26‐loaded microneedles were effective to inhibit the collagen I expression from keloid fibroblasts, demonstrating their potential efficacy for scar treatment.

## 4 DISCUSSION

In recent years, there has been increasing interest to apply biomolecules including peptide/protein as targeted therapeutics. Delivery these molecules by microneedles can avoid their digestions by gastrointestinal enzymes during oral delivery. Previously reported methods to load these biomolecules in the microneedles have been limited. In this paper, we developed a facile, effective, and gentle strategy to load peptide into biocompatible PEGDA microneedles. A model peptide, Gap26 (connexin mimetic peptide) was chosen as a candidate therapeutic and applied on an ex vivo keloid scar model to prove this concept.

To date, PEGDA microneedles have mainly been used to deliver small molecular drugs (e.g., 5‐FU and curcumin) that are chemically stable to high temperature, light, and extreme pH conditions. These stable molecules can be mixed with the monomer before UV exposure or heating to generate microneedles. For example, CPT can reduce collagen biosynthesis, a key step in scar formation.^13^, ^37^ When this drug was encapsulated in PEGDA microneedles through photolithography, no noticeable change was observed in its efficacy to inhibit cell proliferation and collagen I expression (Supporting Information Figures S1, S3, S7).

Subsequently, we loaded hydrophilic sugar and peptide molecules by swelling PEGDA microneedles in an aqueous solution (Figures 1, 2b, and 3c). On hydration, the PEGDA matrix allows solutes to diffuse into the hydrogel matrix. As discussed above, the mesh size of PEG hydrogel,^38^, ^39^ related to its crosslinking density determines whether molecules can readily diffuse into the matrix. Thus, molecules larger than the mesh size cannot be efficiently loaded into PEGDA. Here, we controlled the mesh size of PEGDA microneedles by tuning the UV exposure time during the fabrication and ended up with a PEGDA matrix with a mesh size of 2.0 nm (Supporting Information Figure S4). Thereafter, we tested the loading of various molecules with distinct molecular weights (FITC, FITC‐dextran 4, 10, 20 kDa). Due to the difference in molecular weight, their hydrodynamic sizes vary from angstrom scale to 1.3 to 2.0 to 2.8 nm.^40^ In this case, FITC‐dextran molecules bigger than 10 kDa are larger than the mesh size of PEGDA. Thus, it is expected that minimal quantities of larger molecules were observed in microneedles (Figure 2b,c). Furthermore, Stokes‐Einstein equation indicates diffusion is related with the radius of the solute on a fixed temperature and solvent.^41^, ^42^ So, it is reasonable to observe higher signals for the needles loaded with FITC than FITC‐dextran 4 kDa.

Next, we chose the connexin mimetic peptide, Gap 26 (MW = 1550.8) as a model drug to be loaded into PEGDA microneedles and applied for keloid scar therapy. Inhibition of cell‐cell communications through gap junctional couplings is an emerging concept to treat scar.^28^ Connexin mimetic peptides like Gap 26 are valuable tools to inhibit connexin functions, making it promising for scar reduction.^24^, ^43^ We confirmed Gap26 can suppress the cell‐cell communication between keloid fibroblasts (Figure 3a,b) and loaded Gap 26 into PEGDA microneedles. The loading of Gap26 is similar to that of dextran. As indicated in Fick's law, J=−D∇φ, where *J* is the diffusion flux of the amount per unit area per unit time, *D* is the diffusion coefficient, ∇φ is the concentration gradient. The peptide solution with higher concentration generates bigger concentration gradient, which allows more peptide molecules to diffuse into the polymeric network given a similar time period and surface area. An interesting phenomenon is that FITC and FITC‐dextran 4 kDa were mainly located on the edges of microneedles, while Gap26 exhibited more homogenous distribution after loading. This may be related to their different molecular structures, which requires the detailed further studies. The amount loaded to each tip of microneedles through this strategy can reach 2.52 ± 0.06 μg of Gap26, which is similar to the previously reported coating method.^18^ However, our loading strategy is comparatively more straightforward since formulation viscosity or surface tension does not need adjustment.

After 7 hr, 13.47 ± 4.52 μg of FITC‐Gap 26 was released from microneedles with 252.13 ± 5.94 μg loaded. As the release was detected by immersing the whole patch inside water at 37°C, the swelling occurs throughout the whole patch. As such, released Gap26 from the tips may enter the matrix at the base of microneedles as well, possibly resulting in the relatively low proportion of Gap 26 release. Nonetheless, this situation is not likely to occur in the future therapeutic applications, as skin penetration is limited to microneedle tips.

The efficacy of Gap26‐loaded microneedles for keloid scar treatment was investigated on an ex vivo keloid scar model. Application of Gap 26‐loaded microneedles (especially when applied twice in the treatment period; 4 weeks) could inhibit collagen I expression efficiently, demonstrating the possibility of this peptide loading system in the treatment of keloid scar. This signifies an interesting transdermal drug delivery system, which is potentially for loading other small molecular weight hydrophilic drug molecules in the applications of various diseases.

## 5 CONCLUSIONS

In summary, a facile, effective, and gentle strategy is reported to load peptides into PEGDA microneedles by exploiting the swelling phenomena of PEGDA in aqueous solutions. By regulating UV crosslinking time, the swelling ratio, and mesh size of PEGDA microneedles can be controlled. This allows loading of molecules less than 4 kDa. As a proof of concept, the model peptide, Gap 26 was loaded into the PEGDA microneedles to suppress the gap junction‐based intercellular communication between keloid fibroblasts which leads to reduced collagen I expression in an ex vivo model. This exhibits its therapeutic potentials for keloid scar.

## Supporting information

Additional Supporting Information may be found online in the supporting information tab for this article.

Supporting Tables and Figures
