# Supplementary material for: Peptide delivery with poly(ethylene glycol) diacrylate microneedles through swelling effect
Source: Bioeng Transl Med. 2017 Jul 14;2(3):258–67. doi: 10.1002/btm2.10070 (PMC5689501; doi:10.1002/btm2.10070)
Supplement: Supplementary file 1 — Supporting Tables and Figures [file BTM2-2-258-s001.docx]

Supporting Information for

Peptide Delivery with Poly(ethylene glycol) Diacrylate Microneedles through Swelling Effect

Shiying Liu^a^, David C Yeo^a^, Christian Wiraja^a^, Hong Liang Tey^b,c^, Milan Mrksich^d,e^, Chenjie Xu^a,e^*

^a^ School of Chemical and Biomedical Engineering, Nanyang Technological University, 70 Nanyang Drive, 637457, Singapore;

^b^ National Skin Centre, 1 Mandalay Road, 308205, Singapore;

^c^ Lee Kong Chian School of Medicine, Nanyang Technological University, 50 Nanyang Avenue, 639798, Singapore;

^d^ Department of Chemistry, Northwestern University, 2145 Sheridan Road, Evanston, Illinois 60208, United States;

^e^ NTU-Northwestern Institute for Nanomedicine, Nanyang Technological University, 50 Nanyang Avenue, 639798, Singapore.

Correspondence: Tel (+65) 6513 2893; cjxu@ntu.edu.sg

**Table S1**. Primer sequences for real-time polymerase chain reactions

| Gene | Forward primer sequence (5′ − 3′) | Reverse primer sequence (5′ − 3′) |
| --- | --- | --- |
| GAPDH | ACAACTTTGGTATCGTGGAAGG | GCCATCACGCCACAGTTTC |
| Collagen I | CAGAACGGCCTCAGGTACCA | CAGATCACGTCATCGCACAAC |

**Table S2**. FITC-Gap 26 loading amount and efficiency into PEGDA microneedles by swelling effect.

| Initial amount in solution (µg) | 500 | 250 | 125 | 62.5 |
| --- | --- | --- | --- | --- |
| Initial concentration in solution (µg/µL) | 5 | 2.5 | 1.25 | 0.625 |
| Loaded amount (µg) | 252.13±5.94 | 130.99±15.13 | 51.75±19.85 | 27.81±1.40 |
| Loading efficiency (%) | 50.43±1.19 | 52.40±6.05 | 41.40±15.88 | 44.50±2.25 |

**
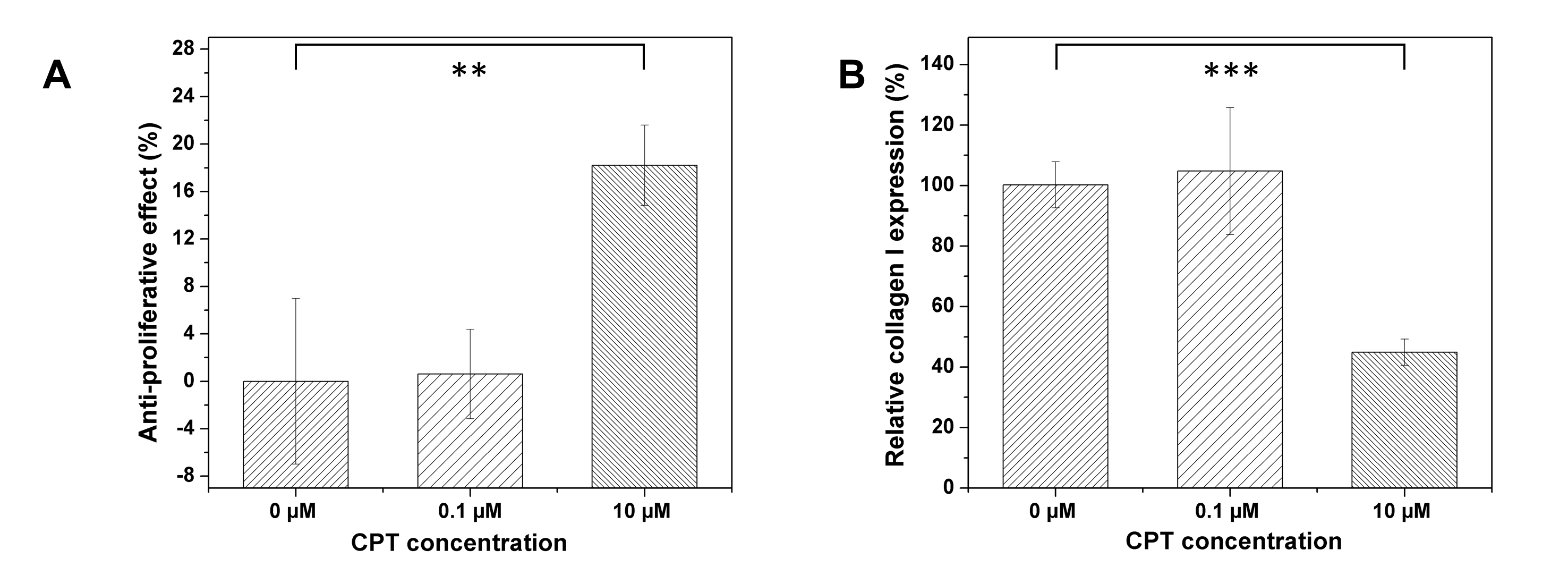
**

**Figure S1**. The inhibition of (A) the proliferation and (B) collagen I expression of keloid fibroblasts by camptothecin (CPT). ** p<0.01, ***p<0.001.


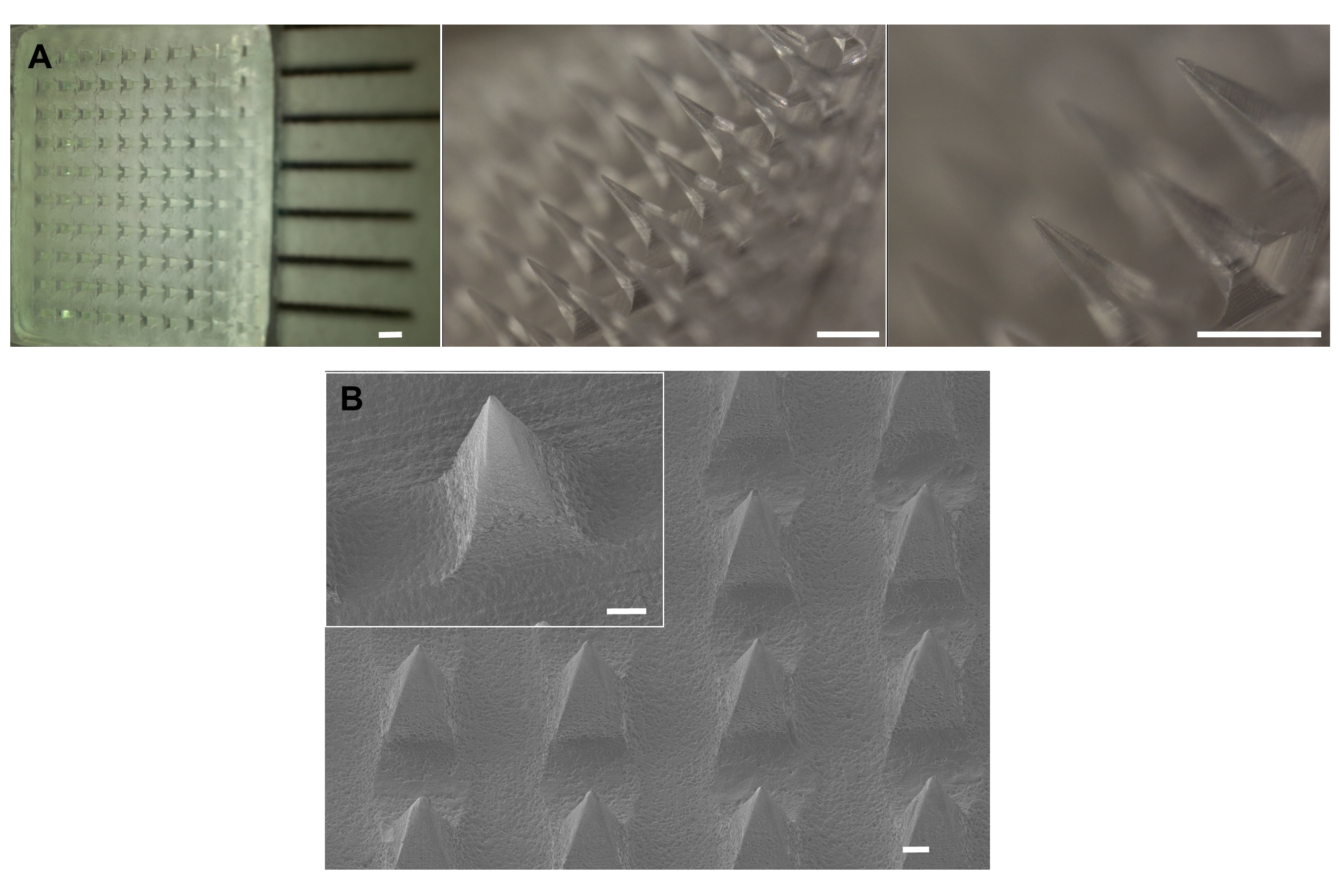


**Figure S2**. (A) Stereomicroscope images of blank PEGDA microneedles, scale bar: 500 μm. (B) Scanning electron microscopy (SEM) images of blank PEGDA microneedles, scale bar: 100 μm.


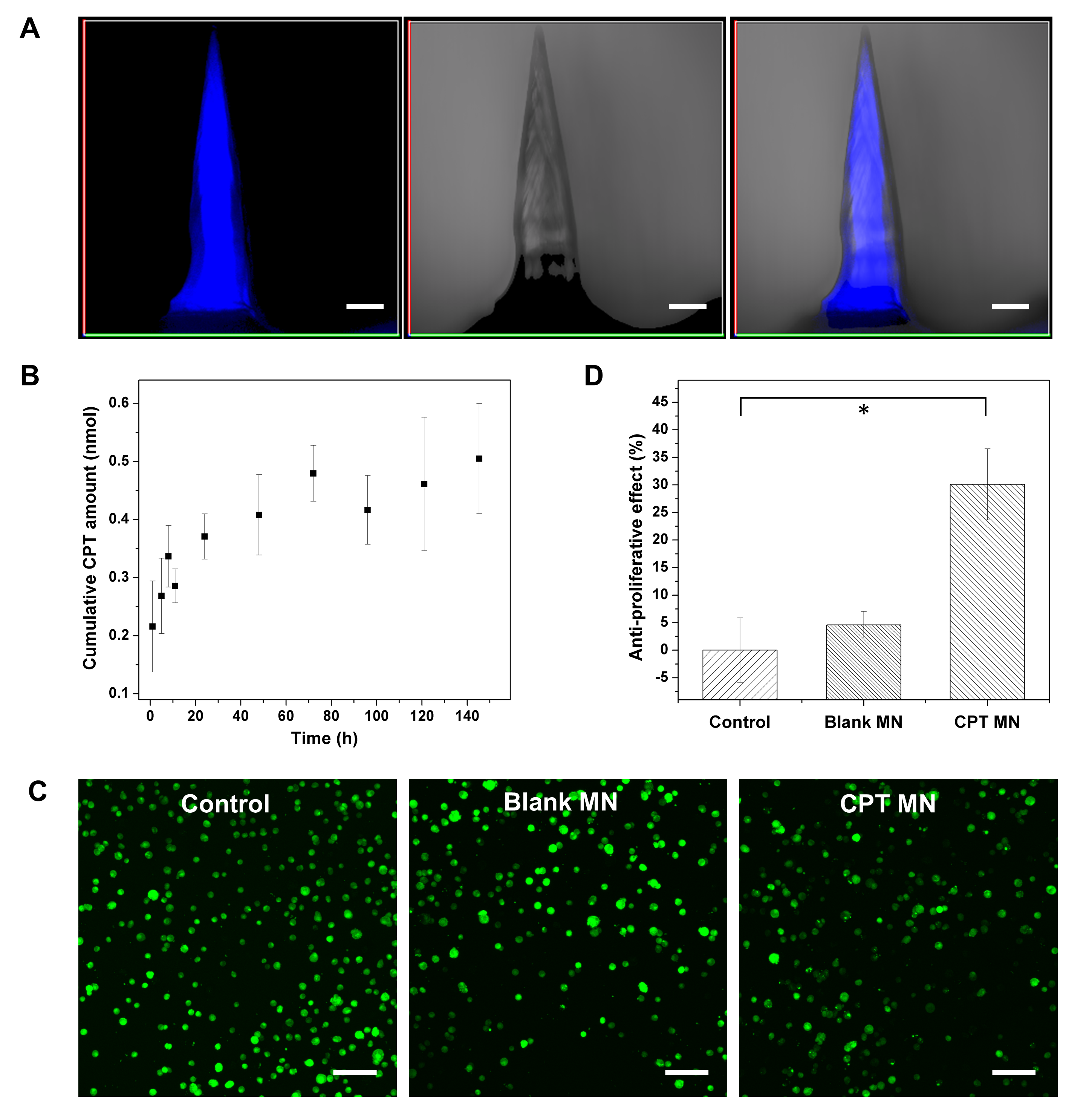


**Figure S3**. (A) Confocal images of CPT loaded microneedles. Scale bar: 100 μm. (B) Release profile of CPT from the microneedle patch in PBS. (C) Confocal images of agarose hydrogel containing keloid fibroblasts after the treatment with PEGDA microneedle patches for 24 h, scale bar: 100 μm. (D) Anti-proliferative effect of CPT loaded microneedles against keloid fibroblasts encapsulated in agarose gel; * p<0.05.


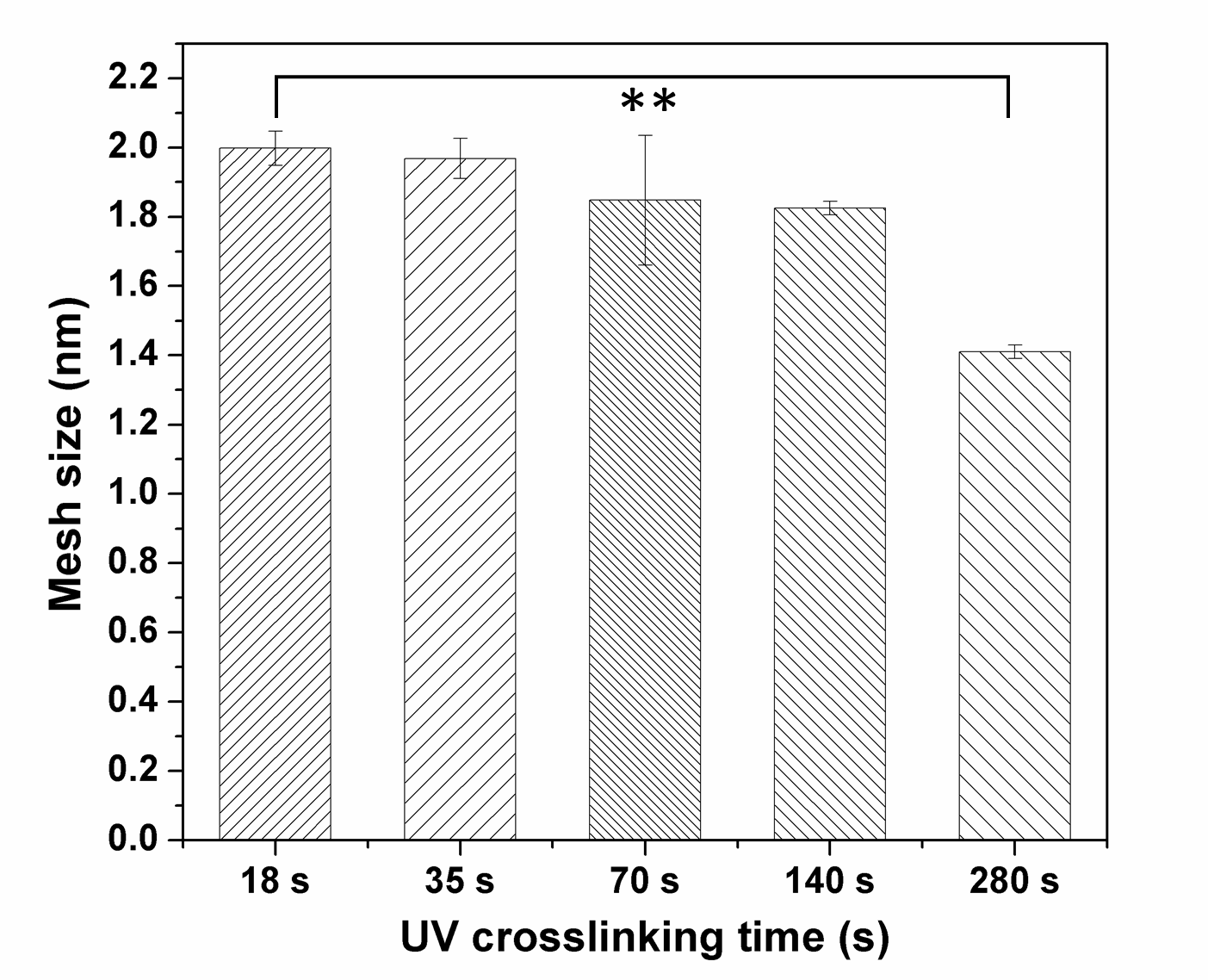


**Figure S4**. Mesh size of PEGDA microneedles prepared with different UV exposure time. ** p<0.01.


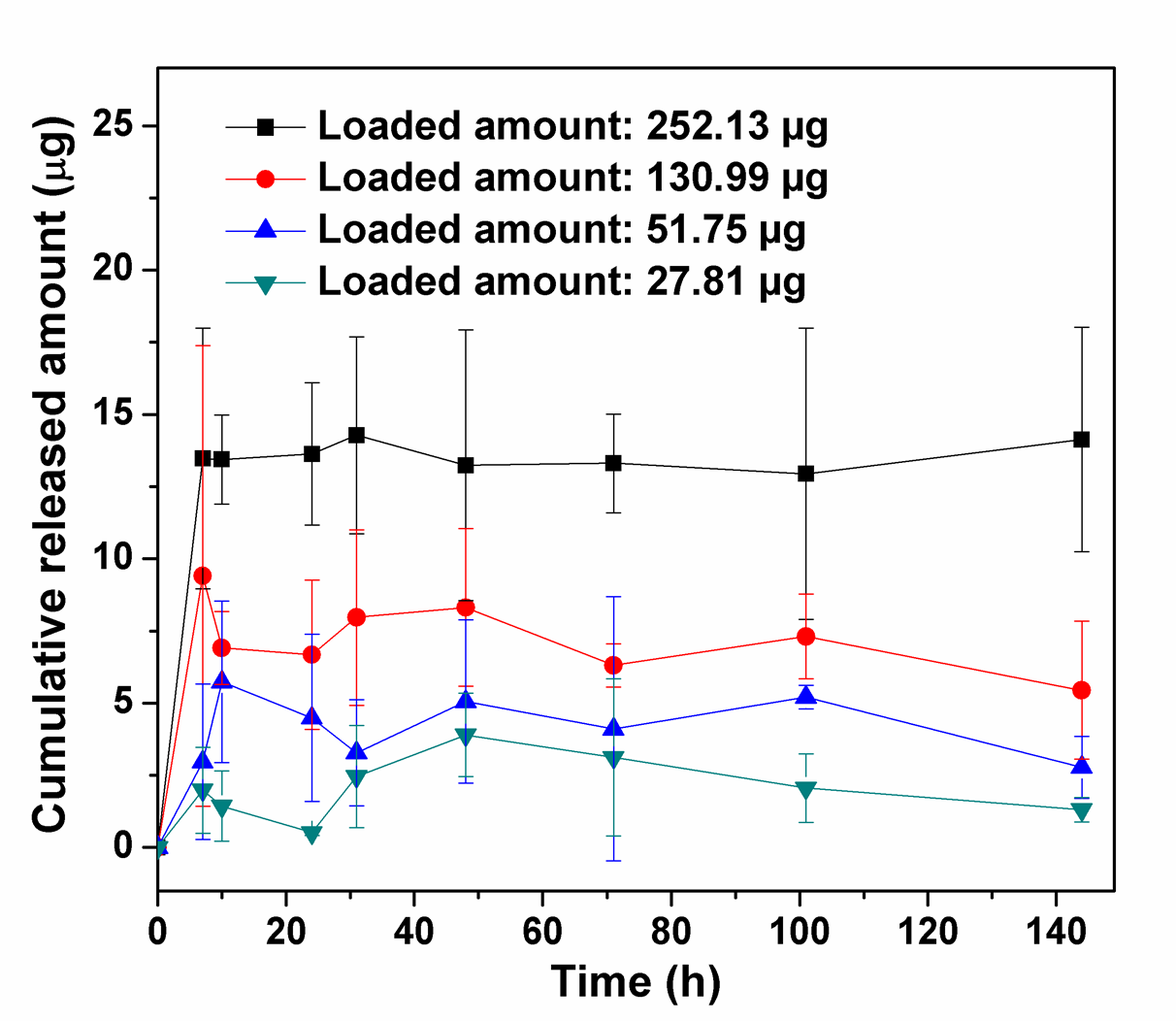


**Figure S5**. Cumulative release of Gap26 from the microneedle patch.


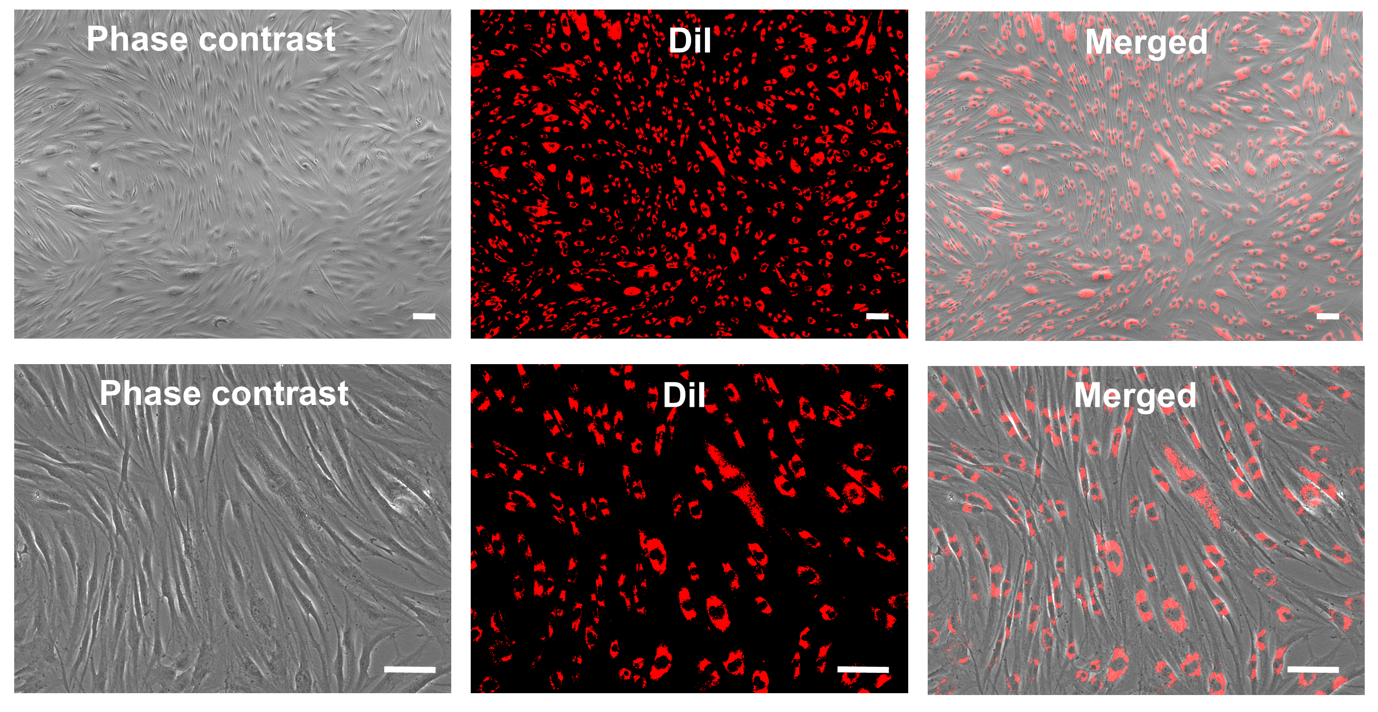


**Figure S6**. Keloid fibroblasts labelled with DiI. scale bar: 100 μm.


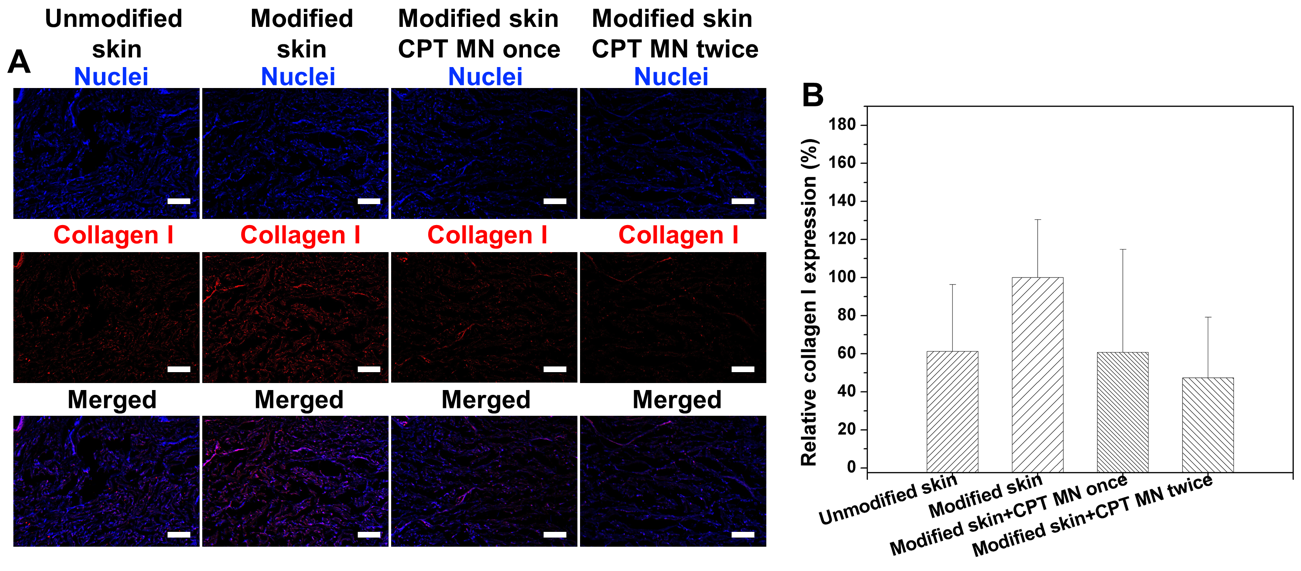


**Figure S7**. (A) Immunostaining of Collagen I expression in the ex vivo keloid model after the treatment with CPT loaded microneedles. Scale bar: 100 μm. (B) Quantitative analysis of collagen I expressions in A by normalization to the keloid model without treatment.


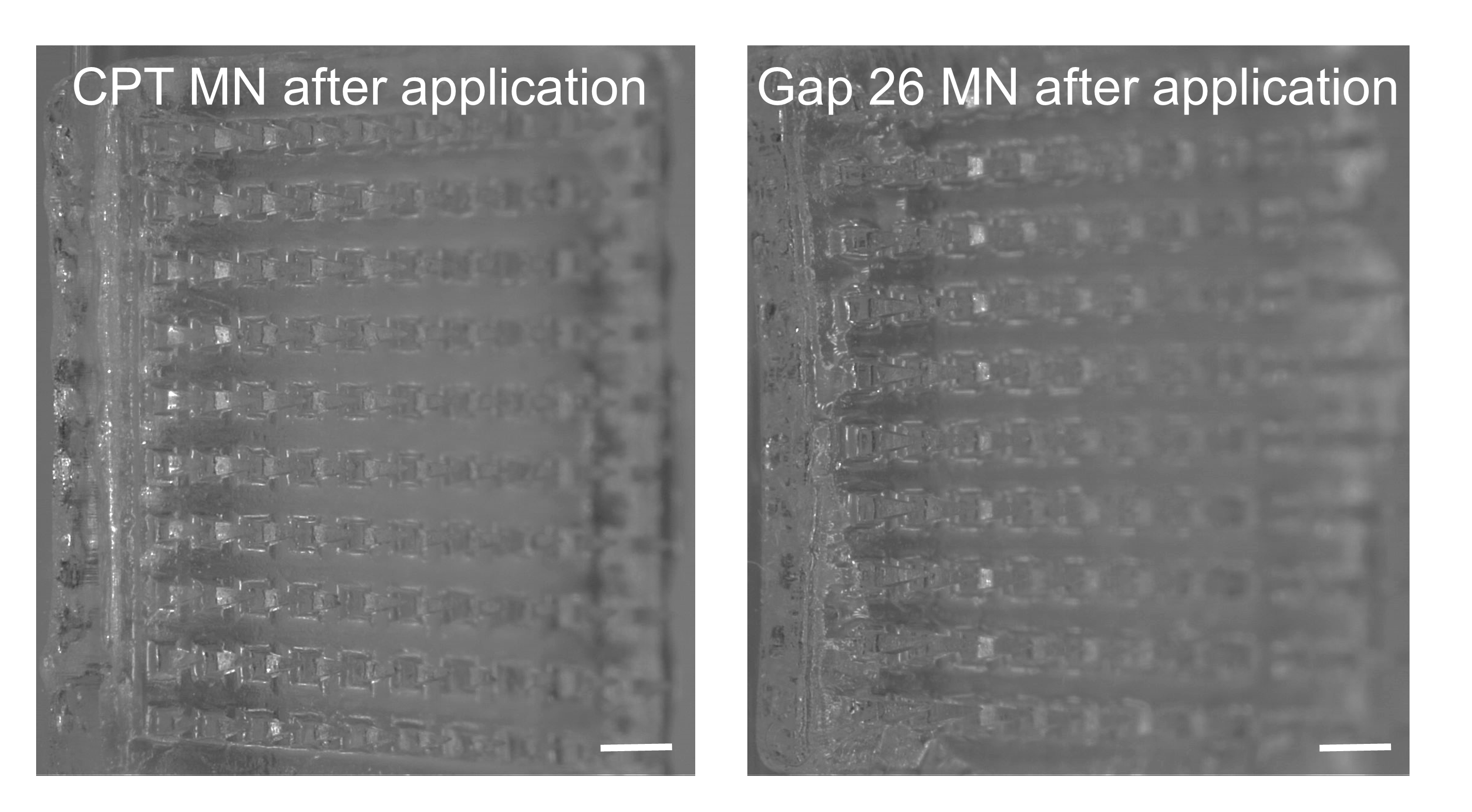


**Figure S8**. Stereomicroscope images of CPT and Gap26 loaded PEGDA microneedles after application in the *ex vivo* skin samples, scale bar: 500 μm.
